# KGTC-DDA:Drug-disease association prediction based on parallel graph isomorphism and transformer networks with cross-attention

**DOI:** 10.1016/j.bbrep.2026.102766

**Published:** 2026-09-03

**Authors:** Jiahao Chen, Zi Liu, Liyi Yu, Wang-Ren Qiu, Weizhong Lin, Xuan Xiao

**Affiliations:** a School of Information Engineering, Jingdezhen Ceramic University, Jingdezhen, Jiangxi, China; b School of Information Engineering, Jingxi Art & Ceramics Technology Institute, Jingdezhen, Jiangxi, China

**Keywords:** Drug-disease association prediction, Multi-feature fusion, Graph isomorphic network, Graph transformer, Cross attention

## Abstract

Drug development is a complex, time-consuming, and costly process, making drug repurposing an important strategy for identifying new therapeutic applications for existing drugs. Computational drug-disease association (DDA) prediction has therefore attracted increasing attention in recent years. Although many existing methods incorporate similarity information and graph neural networks for DDA prediction, effectively capturing both local structural patterns and global dependencies, as well as modeling complex interactions between drugs and diseases, remains challenging. To address these limitations, we propose Drug-disease association prediction model (KNN, GIN, Graph Transformer and Cross-attention, KGTC-DDA), a novel framework that combines Graph Isomorphism Networks (GIN), Graph Transformers (GT), and cross-attention mechanisms for drug-disease association prediction. Specifically, multiple biological similarities, including drug chemical structure similarity, disease phenotype similarity, and Gaussian interaction profile (GIP) kernel similarity, are integrated to construct comprehensive drug and disease similarity graphs. GIN and Graph Transformer modules are then employed in parallel to simultaneously learn local topological features and global contextual representations from the similarity networks. Furthermore, a cross-attention mechanism is introduced to capture high-order interdependencies between drug and disease embeddings, thereby enhancing heterogeneous feature interaction and representation learning. Extensive experiments on three benchmark datasets under 10-times 10-fold cross-validation demonstrate that KGTC-DDA achieves superior predictive performance compared with several state-of-the-art methods in terms of AUROC and AUPR. In addition, experiments on unseen disease prediction further validate the robustness and generalization capability of the proposed framework. These results indicate that KGTC-DDA provides an effective and promising approach for computational drug repositioning and drug-disease association prediction.

## Highlights

• Multi-source similarities capture complementary drug and disease characteristics.
• A parallel GIN-Transformer framework learns structural and relational features.
• Cross-attention models interaction-aware drug-disease representations.
• KGTC-DDA outperforms existing methods on three benchmark datasets.

## 1 Introduction

De novo drug discovery typically proceeds through discovery, preclinical studies, phased clinical trials (I–III), regulatory submission, and eventual approval. This process is characterized by long timelines, substantial cost, and high attrition due to the complexity of biological systems, stringent trial requirements, limited disease understanding, technical constraints, and the specificity demanded of therapeutics. Estimates suggest that discovering a new molecular entity can take 10–15 years and cost tens to hundreds of millions of dollars [1], while success rates remain low [2]. Although pharmaceutical science continues to advance, late-stage failures are common, and overall approval throughput has not dramatically shifted [3,4]. Against this backdrop, drug repurposing—and, in particular, drug–disease association (DDA) prediction—has emerged as a practical strategy to identify new indications for existing drugs, reducing risk, cost, and time to clinic [[5], [6], [7], [8]]Existing DDA approaches fall into four families. Matrix-based methods formulate DDA as a completion problem on a low-rank association matrix, often integrating side information for regularization and interpretability [[9], [10], [11], [12], [13], [14]]. However, they can approach can be limited by overfitting, high computational complexity, challenges in handling highly sparse data, and sensitivity to noise and outliers. Network-propagation methods exploit heterogeneous biomedical networks to diffuse signals across pharmacological and phenotypic spaces [[15], [16], [17]], but their accuracy is tightly coupled to graph completeness and edge quality. Traditional machine learning-based methods reframes DDA as supervised classification using engineered features and similarity kernels [[18], [19], [20]], yet performance hinges on feature quality and negative-sample construction. For example, Gao et al. [18] proposed a Laplace regularized least squares algorithm combined with a similarity kernel fusion method to predict the correlation between drugs and diseases, called DDA-SKF, and Peng et al. [19] proposed a negative sample extraction method based on positive unlabeled learning. In recent years, deep learning has demonstrated powerful representation learning capabilities across molecular, phenotypic, and topological biomedical data, ranging from convolutional neural networks (CNNs) and variational autoencoders (VAEs) to message passing neural networks (MPNNs) and graph neural networks (GNNs) [[21], [22], [23], [24], [25], [26], [27]]. Existing studies have increasingly focused on integrating heterogeneous biological information and exploiting graph-based representation learning strategies for drug-disease association prediction. For example, Li et al. [21] introduced molecular structure and clinical symptoms information into a convolutional neural network framework for DDA prediction. Cai et al. [22] proposed DRHGCN, which integrates heterogeneous network topology information through graph convolutional networks and layer-attention mechanisms. Meng et al. [23] developed DRWBNCF, a weighted bilinear neural collaborative filtering framework capable of modeling complex drug-disease interactions.

More recently, several advanced methods have further improved DDA prediction by incorporating richer biological information and more expressive graph representation learning strategies. For example, REDDA [28] integrated multiple biological relations into a heterogeneous graph neural network framework, enabling more comprehensive information propagation across heterogeneous biomedical entities. Deep Multiple Instance Learning on Heterogeneous Graph for Drug–Disease Association Prediction [29] further improved prediction performance by explicitly modeling heterogeneous drug–disease association structures and learning discriminative representations from heterogeneous graph topology. In addition, Empowering Graph Neural Network-Based Computational Drug Repositioning with Large Language Model-Inferred Knowledge Representation [30] incorporated semantic knowledge inferred from large language models into graph-based drug repositioning frameworks, enabling the model to learn more informative and semantically enriched node representations. And transformer-based heterogeneous graph learning methods have demonstrated strong capability in drug–disease association prediction. Recently, Liu et al. [31] proposed AMDGT, a dual Graph Transformer framework combined with heterogeneous message passing mechanisms to model complex interactions between drugs and diseases, achieving strong predictive performance on multiple benchmark datasets.

Although these methods have achieved promising performance, several limitations remain. First, many existing approaches primarily focus on either local neighborhood aggregation or global dependency modeling, while lacking an effective mechanism to jointly capture both local topological patterns and long-range structural dependencies. Second, heterogeneous interactions between drug and disease representations are often insufficiently modeled, limiting the ability to capture complex cross-domain relationships. Third, some heterogeneous graph-based methods heavily rely on explicit association propagation, which may introduce noisy information propagation when known drug–disease associations are sparse or incomplete.

To address these limitations, we propose KGTC-DDA, a novel drug-disease association prediction framework based on parallel graph representation learning and bidirectional cross-attention. Firstly, integrated drug and disease similarity networks are first constructed from multi-source biological data, followed by K-nearest neighbor (KNN) densification to alleviate sparsity. Then, Graph Isomorphism Networks (GIN) and Graph Transformers (GT) are employed in parallel to simultaneously learn local structural features and global contextual dependencies from the similarity graphs. Furthermore, a bidirectional cross-attention mechanism is introduced to explicitly model heterogeneous interactions and mutual dependencies between drug and disease representations.

## 2 Materials and methods

### 2.1 Datasets

In this study, three benchmark datasets were used to evaluate the performance of our models. These datasets are widely used in Drug-disease association prediction tasks and provide information about drugs, diseases, and their known drug-disease associations. The first dataset, Fdataset, is a standard dataset and was constructed by Gottlieb et al. [32], including 593 drugs, 313 diseases, and 1933 verified drug-disease associations. The second dataset, Cdataset, proposed by Luo et al. [15], includes 663 drugs, 409 diseases, and 2532 known associations. The third dataset, LRSSL [33], consist of 3051 verified drug-disease associations involving 763 drugs and 681 diseases. In these datasets, drugs information was obtain from the DrugBank database [34], while diseases are collected from the Online Mendelian Inheritance in Man (OMIM) database [35]. In this study, due to the observation of the databases used in previous studies, the drug DB00510 is synonymous with the drug DB00313 in DrugBank, Therefore Fdataset was preprocessed to account for this redundancy before analysis. The statistics of the three benchmark datasets after preprocessing are summarized in Table 1.

**Table 1:** Statistics of three benchmark datasets.

Dataset	No.of drugs	No.of diseases	The known associations	Sparsity
Fdataset	592	313	1931	0.0104
Cdataset	663	409	2532	0.0093
LRSSL	763	681	3051	0.0059

### 2.2 Similarity calculation

#### 2.2.1 Drug chemical structure similarity

Drugs are typically characterized by various biological and chemical property signatures. A common strategy is to encode drugs as binary feature vectors, where each element denotes the presence or absence of a specific attribute (e.g., chemical substructure). These feature vectors can be converted into different representations, such as chemical fingerprints, from which similarity measures are subsequently calculated. Specifically, we encode drug chemical structural similarity using the Simplified Molecular Line Input System (SMILES) format [36], generate its 2D chemical fingerprint using the Chemical Development Kit (CDK) [37], and then the similarity scores between drugs are calculate using Tanimoto index. The resulting drug chemical structure similarity is denoted as a matrix ${DR}_{s}\in R^{M\times M}$, where M represents the number of drugs.

#### 2.2.2 Disease phenotype similarity

Phenotypic similarity quantifies the degree of resemblance between diseases based on their observable characteristics. It is typically derived through text mining analysis of medical information from the Online Mendelian Inheritance in Man (OMIM) database. Specifically, MinMiner [38] was employed to compute disease phenotypic similarity score. The resulting phenotypic similarity matrix is denoted as ${DS}_{p}\in R^{N\times N}$, where N represents the number of diseases.

#### 2.2.3 Gaussian Interaction Profile (GIP) kernel similarity for drugs and diseases

To complement the structure and phenotypic similarity information, which may be incomplete or insufficient, we introduced Gaussian Interaction Profile (GIP) kernel similarity to mitigate data sparsity and information loss. This approach is based on the assumption that drugs associated with the same disease tend to exhibit similarity. Accordingly, the GIP kernel similarity between drugs is represented as follows.

(1) $${DR}_{g}\left({dr}_{i},{dr}_{j}\right)=\exp\;\left(-\gamma_{dr}\|\;F\left({dr}_{i}\right)-F\left({dr}_{j}\right){\|}^{2}\right)$$

(2) $$\gamma_{dr}=\frac{\gamma_{dr}^{\prime }}{\frac{1}{M}\sum_{i=1}^{M}\|\;F\left({dr}_{i}\right)\;{\|}^{2}}$$

where ${DR}_{g}$ ∈$R^{M\times M}$, F (${dr}_{i}$), F (${dr}_{j}$) are the vectors of the i-th and j-th rows of the drug in the drug-disease association matrix, representing the characteristic vectors of different drugs. $\gamma_{dr}$ is the equilibrium parameter of the drug kernel. $\gamma_{dr}^{\prime }$ is consistent with previous studies [39] and is set to 1.

Similarly, the GIP kernel similarity between diseases is also denoted as.

(3) $${DS}_{g}\left({ds}_{i},{ds}_{j}\right)=\exp\;\left(-\gamma_{ds}\|\;F\left({ds}_{i}\right)-F\left({ds}_{j}\right){\|}^{2}\right)$$

(4) $$\gamma_{ds}=\frac{\gamma_{ds}^{\prime }}{\frac{1}{N}\sum_{i=1}^{N}\|\;F\left({ds}_{i}\right)\;{\|}^{2}}$$

Where ${DS}_{g}$ ∈$R^{N\times N}$, F (${ds}_{i}$), F (${ds}_{j}$) represent the vectors of the disease columns i and j in the drug-disease association matrix, representing the interaction profile of different diseases, $\gamma_{ds}$ is the balance parameter of the disease kernel, and $\gamma_{ds}^{\prime }$ is set to 1.

#### 2.2.4 Calculate the comprehensive similarity of drugs and diseases

As mentioned above, multiple methods were employed to compute similarities between drugs and diseases. To obtain a more reliable and informative measure, we integrated different similarity sources to construct a comprehensive similarity matrix between drugs and diseases. The integration procedure is defined as follows.

(5) $${DR}_{R}\left({dr}_{i},{dr}_{j}\right)=\left\{ \begin{array}{c} \frac{{DR}_{s}\left({dr}_{i},{dr}_{j}\right)+{DR}_{g}\left({dr}_{i},{dr}_{j}\right)}{2},\text{if}\;{DR}_{s}\left({dr}_{i},{dr}_{j}\right)\neq \tau \\ {DR}_{g}\left({dr}_{i},{dr}_{j}\right),\text{otherwise} \end{array}\right.$$

(6) $${DS}_{S}\left({ds}_{i},{ds}_{j}\right)=\left\{ \begin{array}{c} \frac{{DS}_{p}\left({ds}_{i},{ds}_{j}\right)+{DS}_{g}\left({ds}_{i},{ds}_{j}\right)}{2},\text{if}\;{DS}_{p}\left({ds}_{i},{ds}_{j}\right)\neq \tau \\ {DS}_{g}\left({ds}_{i},{ds}_{j}\right),\text{otherwise} \end{array}\right.$$

Among them, ${DR}_{R}\left({dr}_{i},{dr}_{j}\right)\in R^{M\times M}$, ${DS}_{S}$ (${ds}_{i},{ds}_{j})\in R^{N\times N}$ are the integrated drug and disease similarity matrices respectively. ${DR}_{s}$ and ${DR}_{g}$ is the chemical structure similarity and Gaussian interaction profile (GIP) kernel similarity, Similarly, ${DS}_{p}$ and ${DS}_{g}$ represents the phenotype similarity and GIP-based similarity. Each entry in these matrices represents the pairwise similarity score between two drugs or two diseases.

To effectively integrate heterogeneous similarity sources, a threshold-based fusion strategy is adopted. Specifically, when the chemical structure similarity (or phenotype similarity) between two nodes is greater than or equal to a predefined threshold τ , the corresponding biological similarity and Gaussian interaction profile (GIP) similarity are averaged. Otherwise, the GIP similarity is used as a complementary similarity source. In this study, the threshold τ was empirically set to 0.05, was selected based on preliminary experiments to ensure stable similarity integration performance across datasets. This strategy improves robustness by preventing extremely small similarity values from dominating the integration process. Finally, the constructed drug and disease similarity networks are denoted as HR and HD, respectively, which are used for subsequent graph-based representation learning.

### 2.3 Model architecture

After integrating the drug and disease similarity matrices, a k-nearest neighbor (KNN) strategy was employed to construct sparse similarity networks. For each drug (or disease) node, the k nodes with the highest similarity scores were selected as its neighbors. The corresponding edges were retained to generate the drug similarity graph HR ​and disease similarity graph HD for subsequent graph representation learning.

**Fig. 1:**
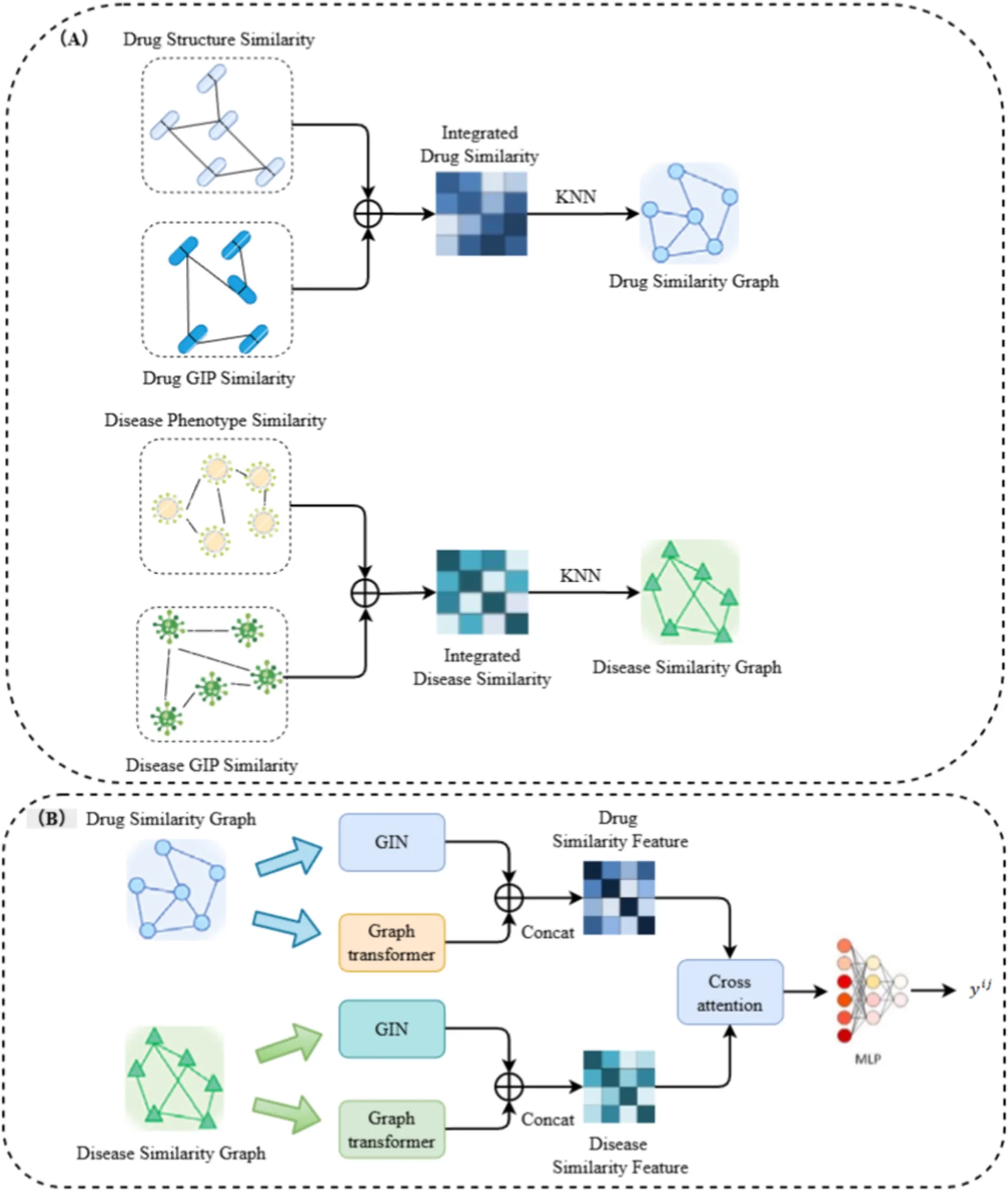
The overall architecture of KGTC-DDA. (A) Feature Fusion, (B) Network structure.

Based on the comprehensive similarity networks constructed from the integrated drug and disease similarities, we designed a prediction framework capable of effectively capturing both local and global relationships within the graph structure (Fig. 1).

The Graph Isomorphism Network (GIN) is utilized to learn topological features from the similarity graphs. As a powerful graph neural network, GIN aggregates neighborhood information to produce node-level and graph-level representations with strong discriminative ability. In parallel, the Graph Transformer module—integrating the Transformer architecture into graph representation learning—captures long-range dependencies and global contextual relationships through the self-attention mechanism.

Both GIN and Graph Transformer modules independently extract features from the drug similarity graph and disease similarity graph. The outputs of these two modules are concatenated to form the drug similarity feature and disease similarity feature, respectively.

To further enhance feature interaction between drugs and diseases, a Cross-Attention (CA) mechanism is applied to align and fuse the heterogeneous features, enabling the model to learn more informative joint representations. Finally, the fused features are fed into a multi-layer perceptron (MLP) for prediction of potential drug–disease associations.

#### 2.3.1 Graph isomorphic networks

Graph Isomorphism Network (GIN) is a neural network architecture specifically designed for processing graph data. Although traditional graph neural networks (GNNs) work well in node classification and graph classification tasks, they have limitations in processing complex graph structures. GIN draws on the theoretical basis of the Weisfeiler-Lehman (WL) test and proposes a new aggregation mechanism, that is, in each layer, the node's neighbor features are weighted summed and combined with a multi-layer perceptron (MLP) to update the node's representation. Through multi-layer information propagation, the node can gradually obtain the structural information of its surrounding neighbors and the entire graph. For drug and disease similarity networks, we use GIN to extract local feature representations of drugs and diseases, and update node features through learnable neighbor aggregation and self-loop. The feature update rules of GIN are as follows.

(7) $$\left\{ \begin{array}{c} {HR}_{r}^{\left(l\right)}={MLP}^{\left(l\right)}\left(\left(1+\epsilon^{\left(l\right)}\right)\cdot {HR}^{\left(l-1\right)}+A_{r}{HR}_{r}^{\left(l-1\right)}\right)\\ \; \end{array}\right.$$

(8) $$\{{HD}_{d}^{\left(l\right)}={MLP}^{\left(l\right)}\left(\left(1+\epsilon^{\left(l\right)}\right)\cdot {HD}^{\left(l-1\right)}+{A_{d}HD}_{d}^{\left(l-1\right)}\right)$$

In the above formulation, ${HR}^{\left(l-1\right)}$ and ${HD}^{\left(l-1\right)}$ denote the node feature matrices of drugs and diseases at the ((l-1))-th GIN layer, where (M) and (N) represent the numbers of drugs and diseases, and (d) denotes the feature dimension. ${HR}_{r}^{\left(l\right)}$ and ${HD}_{d}^{\left(l\right)}$ are the feature matrices of the disease and drug at the lth layer.

The initial embeddings are constructed from the interaction profile feature matrix described in Previous text, which encodes the interaction profiles between drugs and diseases, it is first projected into a unified latent space through a linear transformation layer to ensure dimensional consistency with the input of the GIN network.

The GIN layer then updates node representations by aggregating information from neighboring nodes based on adjacency matrices $A_{r}$ and $A_{d}$, The prediction module is implemented as a three-layer multilayer perceptron (MLP). ${MLP}^{\left(l\right)}$ contains linear transformation and RELU activation. The input dimension corresponds to the concatenated drug and disease embeddings, the final output layer produces a scalar score indicating the probability of drug - disease association, the dropout rate is set to 0.3. $\epsilon^{\left(l\right)}$ is a learnable parameter that controls the relative weight of the node's own features and neighbor features.

#### 2.3.2 Graph transformer

Graph Transformer (GT) is a model that combines the Transformer architecture with a graph neural network, capturing global dependencies in the graph structure through a self-attention mechanism. Compared with traditional graph convolutional networks (GCN) or graph isomorphism networks (GIN), GT can more flexibly model long-distance interactions between nodes and is particularly suitable for processing complex graph data. For the drug and disease similarity network, we use the graph transformer to extract feature representations of drugs and diseases as input to the subsequent prediction model. The following is the detailed update process of GT, taking drugs as an example, where the initial features of the nodes are represented by the similarity matrix ${HR}_{r}$.

(9) $$h^{0}=W^{0}{HR}_{r}+b^{0}$$

(10) $$Q_{ij}=Q^{r}h_{i}^{0},K_{ij}=K^{r}h_{j}^{0},V_{ij}=V^{r}h_{j}^{0}$$

(11) $$A_{k}^{r}\left(v_{i},v_{j}\right)=softmax\left(\frac{Q_{ij}^{r}\cdot \;K_{ij}^{r}}{\sqrt{d_{k}}}\right)V_{ij}^{r}$$

Among them, $h^{0}$ is the input feature, $W^{0}$ and $b^{0}$ are learnable parameters, $d_{k}$ represents the dimension of each head, and $A_{k}^{r}$ represents the multi-head attention score. The final output can be calculated as follows.

(12) $$h_{i}^{L}=h_{i}+P_{k}\left({Concat}_{r=1}^{H}\sum_{j\in M_{i}}A_{k}^{r}\left(v_{i},v_{j}\right)\right)$$

Where $P_{k}$ represents the learnable matrix, $h_{i}^{L}$ is the final node representation of the (l+1)th layer after multi-head attention, and the feature matrices generated by the Graph Transformer are denoted as ${HR}_{t}$ and ${HD}_{t}$, where the Subscript t indicates features extracted by the Graph Transformer. the drug similarity feature matrix is represented as ${HR}_{t}$. Similarly, the disease similarity feature matrix is represented as ${HD}_{t}$.

#### 2.3.3 Cross-attention

To enable interaction modeling between drug and disease representations, the output feature dimensions of (HR) and (HD) are projected into a shared latent space with identical dimensionality (d). Although the original drug–drug similarity matrix (DR) and disease–disease similarity matrix (DS) are defined in different spaces, a learnable linear transformation is applied to map them into a unified feature space before being fed into the cross-attention module. This design ensures that both drug and disease embeddings are comparable in the same representation space, which is necessary for computing attention scores.

After feature extraction through the GIN and Graph Transformer modules, the drug features and disease features are concatenated separately to obtain the final drug representation $H_{r}$ and disease representation $H_{d}$. To further capture high-order dependencies and heterogeneous interactions between drugs and diseases, a Multi-Head Cross-Attention (MHCA) mechanism is employed. Specifically, KGTC-DDA adopts a bidirectional cross-attention strategy, including drug-to-disease attention and disease-to-drug attention, to model mutual dependencies between the two modalities.

For the drug-to-disease attention, given the drug feature matrix $H_{r}\in R^{M\times d}$ and disease feature matrix $H_{d}\in R^{N\times d}$, where M and N denote the number of drug nodes and diseases respectively, In the multi-head cross-attention mechanism, the query, key, and value projections share the same input dimensionality (d), and are mapped into a lower-dimensional subspace ($d_{h}$) through independent learnable matrices ($W_{i}^{Q}$, $W_{i}^{K}$, $W_{i}^{V}$). This design follows the standard transformer architecture, ensuring compatibility of attention computation across different modalities. Before applying cross-attention, drug and disease embeddings are projected into a shared latent space with identical feature dimension (d).

(13) $$Q_{i}=H_{r}W_{i}^{Q}$$

(14) $$K_{i}=H_{d}W_{i}^{K}$$

(15) $$V_{i}=H_{d}W_{i}^{V}$$

Among them, $W_{i}^{Q}$, $W_{i}^{K}$, $W_{i}^{V}\in R^{d\times d_{h}}$ are learnable weight matrices, $d_{h}$ is the feature dimension of each head, and h is the number of heads.

Then, in each head, the dot product of the query $Q_{i}$ and the key $K_{i}$ is calculated to get the attention score, which is normalized by softmax.

(16) $$\text{Attention}\left(Q_{i},K_{i},V_{i}\right)=\text{softmax}\left(\frac{Q_{i}K_{i}^{T}}{\sqrt{d_{h}}}\right)V_{i}$$

Among them, $Q_{i}K_{i}^{T}\in R^{M\times N}$ represents the attention weight of each element to other elements, $\frac{1}{\sqrt{d_{h}}}$ is a scaling factor used to prevent the dot product value from being too large and causing the softmax gradient to be too small, and the output Attention ($Q_{i}$, $K_{i}$, $V_{i}$) is the weighted value vector.

Similarly, disease-to-drug attention is computed by treating disease features as queries and drug features as keys and values. This bidirectional interaction enables the model to simultaneously capture drug-guided disease representations and disease-guided drug representations, thereby enhancing heterogeneous feature interaction and representation capability.

Finally, the outputs of the h heads are concatenated and integrated through linear transformation.

(17) $$\text{MultiHead}\left(H_{r},H_{d}\right)=\text{Concat}\left({head}_{1},{head}_{2}\ldots ,{head}_{h}\right)\;W^{O}$$

Among them, ${head}_{i}=$ Attention ($Q_{i},K_{i}$, $V_{i}$), $W^{O}$ is a learnable weight matrix, and the output MultiHead ($H_{r},H_{d}$) maintains the same dimension as the input. Compared with a unidirectional attention mechanism, the proposed bidirectional cross-attention strategy allows the model to capture complementary interaction patterns from both drug and disease perspectives, thereby improving the expressiveness of heterogeneous representation learning.

### 2.4 Model predictions

The prediction module is implemented as a three-layer multilayer perceptron (MLP). MLP is a feedforward neural network structure consisting of an input layer, several hidden layers, and an output layer. The input layer receives the concatenated drug and disease representations. The hidden layer sizes are set to 128 and 64 neurons, respectively. The first two layers are followed by ReLU activation and a dropout layer with a dropout rate of 0.3 to prevent overfitting. The final output layer consists of a single neuron with a sigmoid activation function, producing the probability of drug–disease association. The MLP parameters were selected based on validation performance and kept consistent across all datasets to ensure fair comparison. The expression of MLP is as follows.

(18) $$h^{l}=Relu\left(W^{l}h^{\left(l-1\right)}+b^{l}\right)$$

where $h^{l}$ is the lth layer output, $W^{l}$ and $b^{l}$ are the weight matrix and the bias vector, respectively, l = 1, 2, …, L represents the number of layers, and Relu is a nonlinear activation function.

To get the final classification result, we concatenate the two embedding vectors $H_{r}$ and $H_{d}$ obtained by multi-head cross attention to represent the drug-disease pair. Specifically, we use a three-layer multi-layer perceptron (MLP) neural network to represent $y_{ij}^{p}$, which represents the probability that the i-th drug is used to treat the j-th disease. The calculation process is.

(19) $$y_{ij}^{p}=\sigma \left({MLP}^{\left(3\right)}\left(\text{Concat}\left(H_{r}^{i},H_{d}^{j}\right)\right)\right)$$

where $\text{Concat}(H_{r}^{i},H_{d}^{j}$) is the concatenation of the i-th drug feature and the j-th disease feature, ${MLP}^{\left(3\right)}$ contains three fully connected layers and ReLU activation, and σ (·) is the sigmoid activation function.

### 2.5 Model optimization

After the above steps, the model learns the final representations of drugs and diseases $H_{r}$ and $H_{d}$. Based on the fact that most of the loss functions used in previous studies are binary cross-entropy (BCE) loss, we use BCE loss as our loss function to handle data imbalance and avoid numerical instability. The loss function is defined as.

(20) $$L_{BCE}=-\frac{1}{M\cdot N}\sum_{i=1}^{M}\sum_{j=1}^{N}\left[{w_{pos}\cdot y}_{ij}\cdot \log\left(y_{ij}^{p}\right)+\left(1-y_{ij}\right)\cdot \log\left(1-y_{ij}^{p}\right)\right]\;$$

Where (i, j) represents the drug–disease pair, $y_{ij}\in \left\{0,1\right\}$ denotes the true association label, M and N represent the numbers of drugs and diseases, and $y_{ij}^{p}$ represents the predicted association probability. Since the number of known drug–disease associations is much smaller than the number of unknown associations, a positive class weighting strategy is adopted to alleviate data imbalance. Specifically, the weighting coefficient $w_{pos}$ is calculated as.

(21) $$w_{pos}=\frac{N_{neg}}{N_{pos}}$$

where $N_{pos}$ and $N_{neg}$ denote the numbers of positive and negative samples, respectively. This weighting strategy increases the contribution of positive associations during optimization and prevents the model from being biased toward negative samples.

## 3 Experiment

### 3.1 Parameter setting

**Fig. 2:**
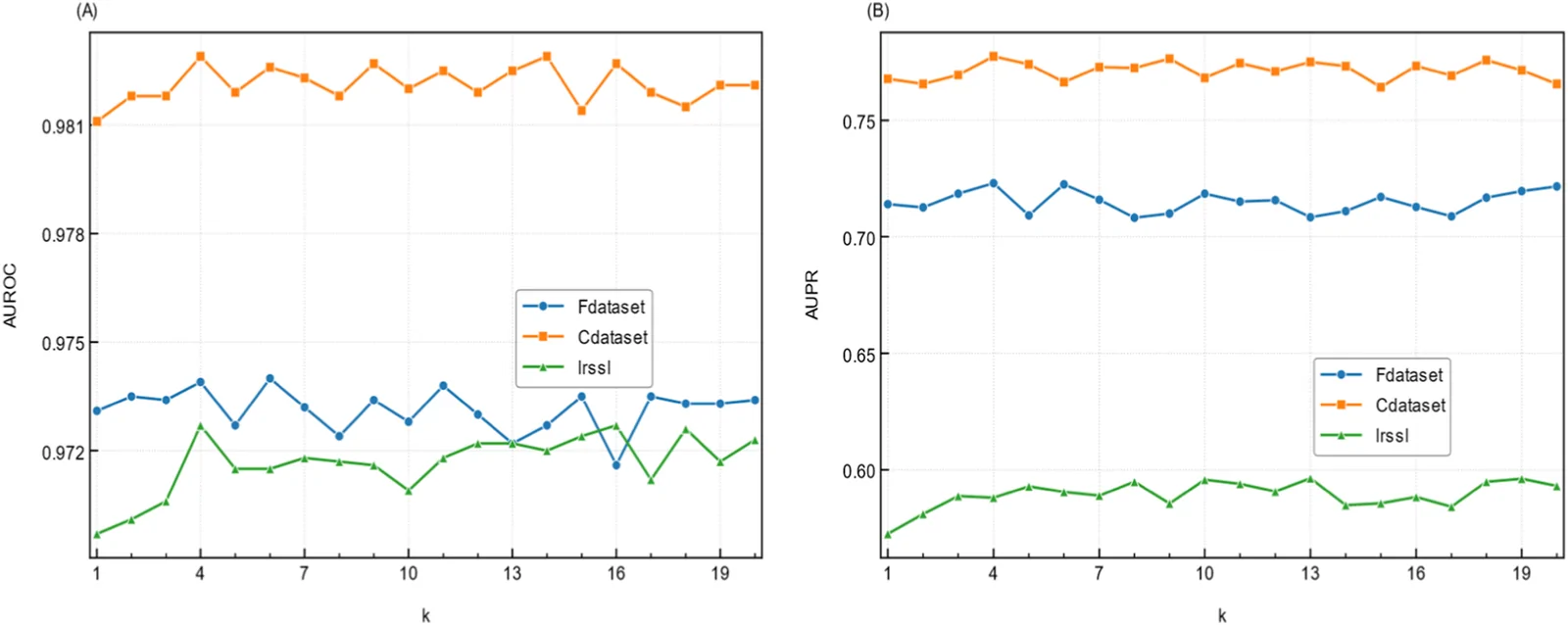
The impact of using different neighbor numbers k on KGTC-DDA. (A) Changes in AUROC. (B) Changes in AUPR.

Regarding KGTC-DDA, several hyperparameters are tuned to optimize model performance, including the total number of training epochs, learning rate, number of neighbors, number of GIN layers, and number of attention heads. The total number of training epoch is selected from {1000, 2000, 3000, 4000}. The parameter k represents the connection between each drug or disease and its k most similar drugs or diseases. However, excessively large k value may generate noise and reduce discriminative power, therefore, the number of neighbors k is considered to be determined in the range of {1, 4, 7, 10, 13, 16, 19}. The AUROC and AUPR values corresponding to different numbers of neighbors k across the three datasets as shown in Fig. 2. Based on the comparative results, the optimal configuration was determined to include 4000 total training epochs, a learning rate of 0.001, and number of neighbors *k* = 4 for subsequent experiments. The main hyperparameters of KGTC-DDA are summarized in Table 2. Hyperparameter selection was performed empirically based on validation experiments to achieve stable predictive performance across benchmark datasets.

**Table 2:** The hyperparameter settings of KGTC-dda.

Parameter	value
Optimizer	Adam
training epoch	4000
Learning rate	1e-3
KNN neighbor	4
Number of GIN layers	2
Transformer heads	4
Output dimension GIN	75
Input dimension	500
Output dimension	75
Attention heads	3
Droupout	0.3

### 3.2 Baseline methods

To evaluate the performance of the model, we compared KGTC-DDA with five state-of-the-art methods on three datasets (Fdataset, Cdataset, LRSSL):

SCMFDD [40]: proposed a matrix decomposition method based on similarity constraints, which introduced the similarity information of drugs and diseases into the matrix decomposition process to enhance the biological consistency of the latent representation, thereby improving the accuracy of drug-disease association prediction.

NIMCGCN [41]: This method uses a graph convolutional network to learn latent feature representations from the similarity network of miRNAs and diseases, and predicts miRNA-disease associations through a neural-induced matrix completion method, realizing an end-to-end supervised learning framework and significantly improving the prediction accuracy.

DRHGCN [22]: This is a GCN-based drug repositioning method which designs inter- and domain feature extraction modules and a layer attention and mechanism.

DRWBNCF [23]: This method combines weighted bilinear graph convolution operations and neural collaborative filtering to integrate drug-disease associations, drug and disease neighborhoods, and their interaction information to capture local structural features. It uses a multi-layer perceptron for prediction, and introduces theα-balanced focal loss function and graph regularization to improve prediction accuracy and model robustness.

DRAGNN [27]: DRAGNN uses a graph attention mechanism to assign weights to heterogeneous nodes and enhance the information aggregation effect of target nodes. This method avoids over-embedding of self-node information, emphasizes the fusion of heterogeneous and homogeneous neighbor information, and uses a multi-layer perceptron for final association prediction, thereby improving prediction accuracy and model generalization.

### 3.3 Evaluation metrics

The known drug-disease pairs were used as positive samples, and the unknown ones were used as negative samples. The positive and negative samples were divided into 10 subsets of approximately the same size, each of which was used as a test set in turn, and the other nine subsets were used as training sets. Two indicators were used to evaluate the performance of the model: the area under the receiver operating characteristic curve (AUROC) and the area under the precision-recall curve (AUPR). It should be noted that when there is an imbalance in the data categories, AUPR is usually more worthy of attention than AUROC [42,43].

## 4 Results and discussions

### 4.1 Performance of KGTC-DDA in 10-fold cross-validation

To comprehensively evaluate the performance and stability of KGTC-DDA, we conducted 10 independent runs of 10-fold cross-validation experiments on three benchmark datasets. The final evaluation results were reported as the mean ± standard deviation of AUROC and AUPR across the 10 independent runs. For fair comparison, all competing models were configured using their optimal hyperparameter settings as reported in the corresponding publications. Model training was performed using the Binary Cross-Entropy (BCE) loss function, and the experimental results are summarized in Table 3.

**Table 3:** The AUROCs and AUPRs of KGTC-DDA and the other five comparative models under 10-times 10-fold cross-validation.

Datasets	SCMFDD	NIMCGCN	DRHGCN	DRWBNCF	DRAGNN	KGTC-DDA
**AUROC**						
Fdataset	0.7740 ± 0.001	0.8214 ± 0.011	0.9441 ± 0.002	0.9247 ± 0.001	0.9441 ± 0.002	**0.9722 **±** 0.002**
Cdataset	0.7937 ± 0.001	0.8273 ± 0.017	0.9604 ± 0.001	0.9406 ± 0.001	0.9478 ± 0.007	**0.9815 **±** 0.001**
LRSSL	0.7668 ± 0.001	0.7775 ± 0.012	0.9573 ± 0.001	0.9359 ± 0.002	0.9504 ± 0.005	**0.9713 **±** 0.002**
Avg	0.7782	0.8087	0.9539	0.9337	0.9475	**0.975**
**AUPR**						
Fdataset	0.0056 ± 0.000	0.1235 ± 0.028	0.5433 ± 0.006	0.2494 ± 0.001	0.5989 ± 0.025	**0.7108 **±** 0.009**
Cdataset	0.0055 ± 0.000	0.1743 ± 0.071	0.6401 ± 0.005	0.3396 ± 0.001	0.6144 ± 0.025	**0.7661 **±** 0.007**
LRSSL	0.0038 ± 0.000	0.0874 ± 0.010	0.4173 ± 0.005	0.1799 ± 0.001	0.4997 ± 0.021	**0.5835 **±** 0.011**
Avg	0.0050	0.1284	0.5335	0.2563	0.5710	**0.6868**

**Fig. 3:**
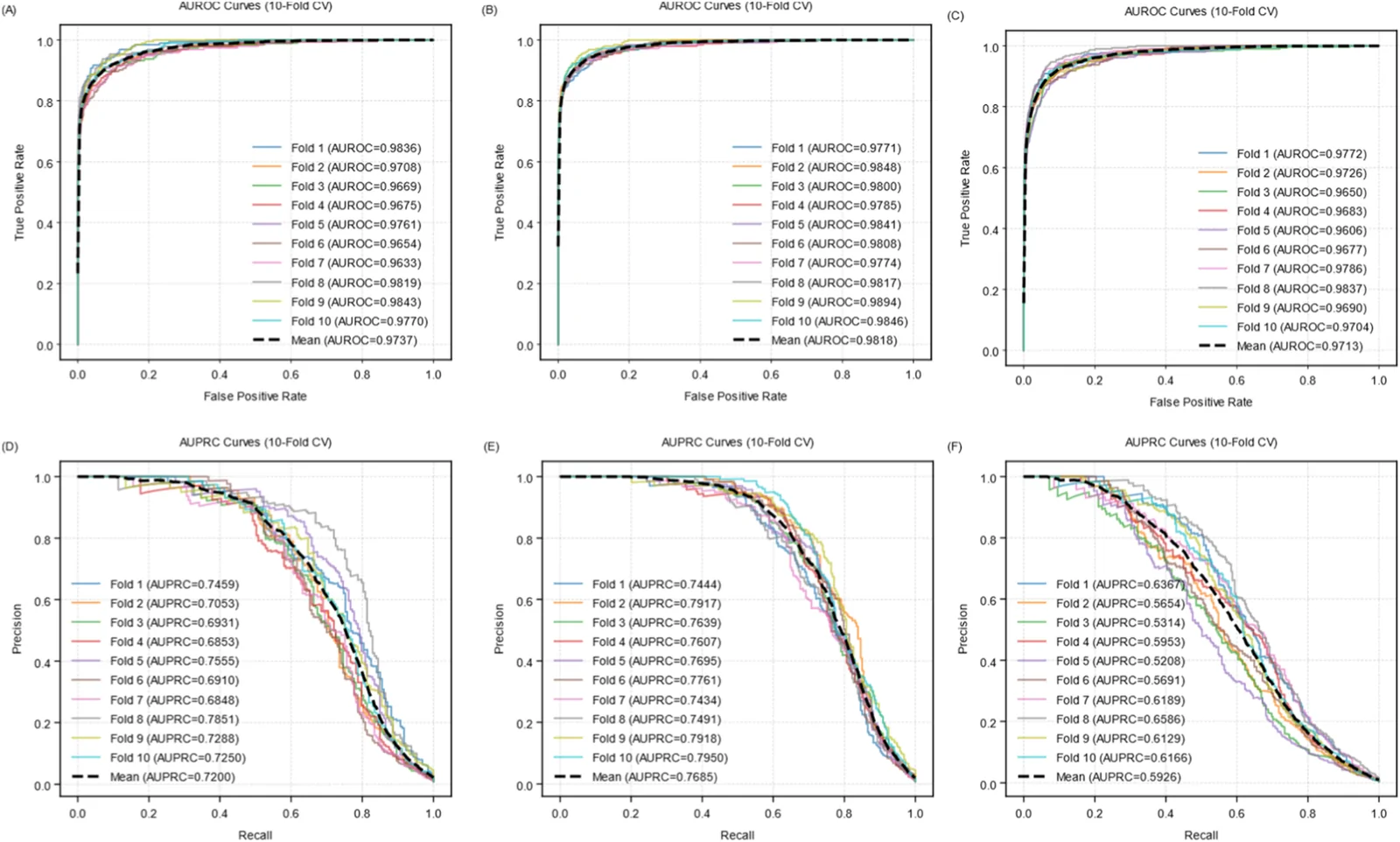
ROC and PR curves obtained from one representative 10-fold cross-validation run on the three benchmark datasets.

According to Table 3, KGTC-DDA achieves the best overall performance compared with five state-of-the-art methods, yielding an average AUROC of 0.9752 and an average AUPR of 0.6930. Relative to the strongest existing baselines, KGTC-DDA improves the AUROC by 2.75% and the AUPR by 11.58%, demonstrating its superior predictive capability. For visualization purposes, Fig. 3 presents the ROC and PR curves obtained from one representative 10-fold cross-validation run on the three benchmark datasets. The results further demonstrate the robustness and generalization capability of KGTC-DDA for drug-disease association prediction.

### 4.2 Predicting drug candidates for new diseases

To further validate the predictive capability of KGTC-DDA for identifying potential drugs associated with new diseases, we conducted an additional experiment on the Fdataset. For each disease, all known drug–disease associations (DDAs) involving that disease were removed and used as the test set, while the remaining associations served as the training data. This experimental setup ensures that the diseases in the test samples are previously unseen, requiring the model to rely solely on similarity-based representations to infer potential therapeutic candidates.

The averaged results across all test sets are summarized in Table 4. Compared with five representative baseline methods, KGTC-DDA achieved the best performance, with an AUROC of 0.9128 and an AUPR of 0.4325. These results demonstrate that KGTC-DDA can effectively generalize to unseen diseases and accurately predict promising drug candidates for novel therapeutic applications.

**Table 4:** AUROCs and AUPRs of all methods in predicting candidates for new diseases on Fdataset.

Methods	AUROC	AUPR
SCMFDD	0.5930 ± 0.015	0.0190 ± 0.025
NIMCGCN	0.6158 ± 0.016	0.0496 ± 0.021
DRHGCN	0.8110 ± 0.014	0.1180 ± 0.010
DRWBNCF	0.7750 ± 0.010	0.1740 ± 0.011
DRAGNN	0.8291 ± 0.009	0.1247 ± 0.011
KGTC-DDA	0.9128 ± 0.005	0.4325 ± 0.023

### 4.3 Ablation experiment

To gain a deeper understanding of the contribution of each component within KGTC-DDA, we conducted ablation experiments on three benchmark datasets. Specifically, three model variants were constructed by removing key modules to assess their respective impacts on overall performance. The variants are defined as follows.

(1) KGTC-DDA w/o GIN: This variant omits the Graph Isomorphism Network (GIN), thereby excluding the feature extraction of neighboring node information.
(2) KGTC-DDA w/o GT: In this variant, the Graph Transformer module is removed, preventing the model from leveraging global structural dependencies in the graph.
(3) KGTC-DDA w/o CA: This variant excludes the Cross-Attention (CA) mechanism, thus eliminating the ability to capture complex interactions between drug and disease features.

Table 5 presents a comparison between the full KGTC-DDA model and its three ablated variants under 10-fold cross-validation. Among the three variants, removing the Cross-Attention module results in the largest decrease in AUPR, suggesting that modeling the interactions between drug and disease representations plays a particularly important role in improving prediction precision. In contrast, removing the GIN or Graph Transformer also degrades the model performance, demonstrating that local neighborhood aggregation and global structural representation are both indispensable for learning informative node embeddings. Although the performance reductions of the ablated models are relatively moderate, this suggests that the three modules provide complementary representations and jointly contribute to a robust prediction framework, rather than relying excessively on any single component.

**Table 5:** The AUROCs and AUPRs of KGTC-DDA and its three variants under 10-fold cross-validation.

Variants	Fdataset		Cdataset		LRSSL	
	AUROC	AUPR	AUROC	AUPR	AUROC	AUPR
KGTC-DDA w/o GIN	0.9712	0.7154	0.9802	0.7595	0.9634	0.5233
KGTC-DDA w/o GT	0.9684	0.6970	0.9800	0.7535	0.9693	0.5624
KGTC-DDA w/o CA	0.9719	0.6998	0.9812	0.7503	0.9622	0.5135
KGTC-DDA	0.9724	0.7206	0.9822	0.7689	0.9712	0.5896

It is worth noting that the performance differences between the complete model and each ablated variant are relatively small. This observation suggests that the three modules collaboratively contribute to feature learning and that the removal of any single module can be partially compensated by the remaining components. Such complementary behavior indicates that KGTC-DDA possesses a certain degree of robustness rather than relying excessively on a single network component. Nevertheless, the consistent superiority of the complete model across all benchmark datasets confirms that integrating local structural learning (GIN), global contextual modeling (Graph Transformer), and cross-modal interaction learning (Cross-Attention) is beneficial for improving prediction performance.

### 4.4 Attention visualization

To enhance the interpretability of the model, we visualize the cross-attention. Unlike the standard softmax attention mechanism, which typically produces a diffuse weight distribution, we employ sparsemax as the attention activation function. Sparsemax encourages a sparse attention distribution by forcing many irrelevant weights to zero, thus highlighting only the most relevant drug-disease interactions.

**Fig. 4:**
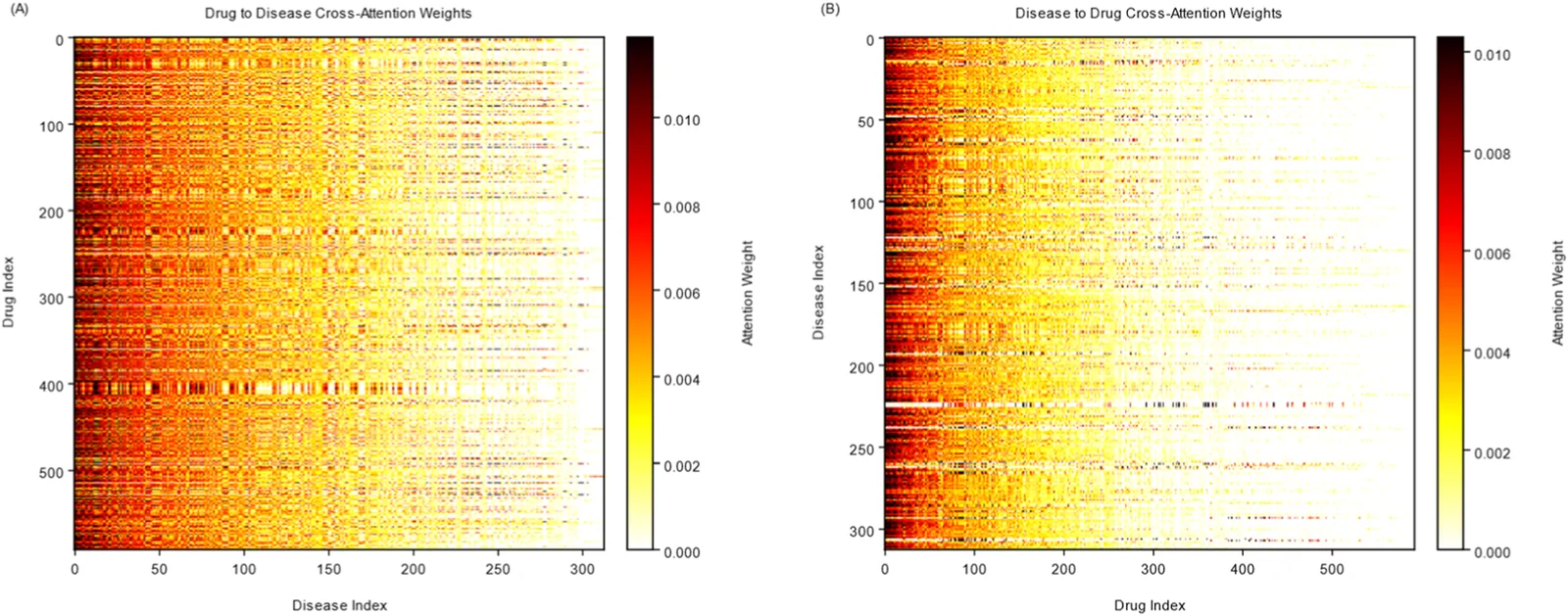
Visualization of attention mechanisms. (A) Drug to Disease attention Weights. (B) Disease to Drug attention Weights.

We also rearranged rows and columns based on the average attention intensity for each drug/disease node to reveal potential clustering patterns. In the drug to disease attention heatmap (Fig. 4A), warmer colors (higher weights) are concentrated in the upper left region, indicating that some drugs strongly focus on specific disease groups. Similarly, the disease to drug attention heatmap (Fig. 4B) exhibits a similar clustering structure, with certain diseases clustered in specific drug subgroups.

Quantitative analysis further supports the sparsity and selectivity of the learned attention. The maximum attention weight in the drug to disease direction reaches 19.1%, and the maximum attention weight in the disease to drug direction reaches 13.0%, while most weights are exactly zero. This sparse pattern suggests that the model does not uniformly focus on all possible interactions, but rather selectively focuses on biologically plausible associations that may correspond to common molecular mechanisms, pathways, or known therapeutic relationships.

### 4.5 Discussion

Although KGTC-DDA achieves competitive predictive performance on benchmark datasets, its performance remains lower than that reported by recent transformer-based heterogeneous graph methods such as AMDGT. One possible reason is that AMDGT explicitly performs heterogeneous message propagation over drug–disease association topology using a dual Graph Transformer architecture, which may provide stronger capability for modeling complex cross-domain interactions.

In contrast, KGTC-DDA primarily learns representations from drug similarity graphs and disease similarity graphs, while heterogeneous interactions are modeled through bidirectional cross-attention rather than explicit heterogeneous graph propagation. Although this design improves representation robustness and reduces potential noise propagation caused by sparse associations, it may limit the model's ability to fully exploit heterogeneous association topology information.

In addition, AMDGT employs deeper transformer-based interaction modeling mechanisms, which may further enhance long-range dependency learning and heterogeneous feature fusion. Therefore, incorporating stronger heterogeneous graph representation learning strategies and more advanced transformer architectures represents an important direction for future improvement of KGTC-DDA.

## 5 Conclusion

In this study, we proposed a novel drug–disease association prediction model, termed KGTC-DDA, which integrates multi-feature fusion and cross-attention mechanisms within a graph neural network framework. The model first constructs multiple similarity matrices to represent drugs and diseases comprehensively. Subsequently, a Graph Isomorphism Network (GIN) and a Graph Transformer are employed to extract local and global structural features, respectively, enabling the model to learn robust representations of drugs and diseases. To further enhance the interaction between the two modalities, a multi-head cross-attention mechanism is introduced. The concatenated feature representations are finally input into a three-layer multilayer perceptron (MLP) to predict drug–disease association probabilities.

Extensive ten-fold cross-validation experiments on three benchmark datasets demonstrate that KGTC-DDA outperforms existing state-of-the-art models in terms of AUROC and AUPR. Moreover, three ablation variants—removing the GIN, Graph Transformer, and Cross-Attention components—were designed to assess their respective contributions. The results confirm that each module plays a vital role in improving model performance. In addition, we visualized the effects of varying the number of neighbors *k* and compared model performance under different configurations.

Despite its strong predictive performance, KGTC-DDA still faces several limitations. First, the current model relies solely on similarity information as input features, without incorporating multi-dimensional biological attributes of drugs and diseases. Second, compared with recent heterogeneous graph transformer-based approaches, the current framework may not fully exploit heterogeneous association topology information due to the absence of explicit heterogeneous message propagation mechanisms. Which might further enhance predictive accuracy. Finally, when predicting associations involving novel drugs or diseases not present in the training data, the similarity features must be reconstructed, limiting generalizability. In future work, we plan to incorporate richer biological information related to drug repositioning, including molecular structures, target proteins, and other heterogeneous biological entities. By integrating diverse data sources and employing more advanced network architectures, we aim to deepen the understanding of the molecular mechanisms underlying drug–disease interactions and improve the robustness and interpretability of predictive models in computational drug discovery. Furthermore, inspired by recent advances in large language model (LLM)-enhanced biomedical representation learning, future studies will explore the integration of LLM-inferred semantic knowledge with graph structural representations to further improve the representation capability, interpretability, and generalization performance of drug–disease association prediction models.

## CRediT authorship contribution statement

**Jiahao Chen:** Conceptualization, Data curation, Formal analysis, Investigation, Methodology, Software, Validation, Visualization, Writing – original draft, Writing – review and editing. **Zi Liu:** Project administration, Supervision, Writing – review and editing. **Liyi Yu:** Data curation, Investigation, Software, Validation, Writing – review and editing. **Wang-Ren Qiu:** Formal analysis, Investigation. **Weizhong Lin:** Resources, Supervision. **Xuan Xiao:** Funding acquisition, Project administration, Resources, Supervision.

## Ethics declaration

The authors have NOT obtained informed consent from participants or their legal representatives. This study involved only secondary computational analysis of existing, non-identifiable data obtained from the COSMIC database. The authors did not directly recruit participants, collect biological samples, or access identifiable personal information. Therefore, additional informed consent from participants was not required for this secondary analysis.

This study was performed in compliance with relevant laws, regulatory frameworks and guidelines where the research took place. Ethics committee approval was not required under relevant laws and institutional guidelines. Ethics committee approval was not required because this study involved secondary analysis of publicly available data obtained from the COSMIC database and did not involve direct recruitment of participants, intervention, or collection of human biological materials.

## Declaration of generative AI and AI-assisted technologies in the manuscript preparation process

During the preparation of this work, the authors used ChatGPT and DeepSeek solely to improve the English language and readability of the manuscript. After using this tool/service, the authors carefully reviewed and edited the content as needed and take full responsibility for the content of the published article.

## Funding

This work was supported by grants from the 10.13039/501100001809National Natural Science Foundation of China (No. 62162032, 32260154, 62562041), 10.13039/501100009102Jiangxi Provincial Department of Education funding for research projects (GJJ2201004, GJJ2400905, GJJ2400902), and Jingdezhen City Science and Technology Plan Project (No.2025JCYJ003).

## Declaration of competing interest

The authors declare no conflict of interests.

## Data Availability

I have shared the link to my data/code.
